# Subacute thyroiditis presenting as simple acute headache was misdiagnosed as meningitis: case report and literature review

**DOI:** 10.1186/s12902-023-01313-6

**Published:** 2023-03-07

**Authors:** Cao Huang, Shuang Shen, Jianping Yao

**Affiliations:** 1 Department of Gastroenterology, Huzhou Central Hospital, Affiliated Central Hospital of Huzhou University, Huzhou, 313000 Zhejiang Province China; 2 School of Medicine, Zhejiang University, Hangzhou, 310003 China; 3 Department of Neurology, Huzhou Central Hospital, Affiliated Central Hospital of Huzhou University, Huzhou, 313000 Zhejiang Province China; 4 Department of Endocrinology, Huzhou Central Hospital, Affiliated Central Hospital of Huzhou University, Huzhou, 313000 Zhejiang Province China

**Keywords:** Subacute thyroiditis, Thyrotoxicosis, Headache, Misdiagnosis

## Abstract

**Background:**

The relationship between headache and thyrotoxicosis has been occasionally mentioned in case reports, but there are few related reports. Thus, the relationship cannot be determined. Few cases of subacute thyroiditis (SAT) presenting as simple headache have been reported.

**Case presentation:**

This case report describes a middle-aged male patient who came to our hospital with acute headache for 10 days. He was initially misdiagnosed as meningitis due to headache, fever, and increased C-reactive protein. Routine antibacterial and antiviral therapy did not improve his symptoms. Blood test suggested thyrotoxicosis, and color ultrasound suggested SAT sonography. He was diagnosed with SAT. With the treatment of SAT, the headache was relieved after the thyrotoxicosis improved.

**Conclusion:**

This patient is the first detailed report of SAT presenting with simple headache, which is helpful for clinicians to differentiate and diagnose atypical SAT.

## Background

Subacute thyroiditis (SAT) is a self-limiting disease with common clinical manifestations of neck pain spreading to the jaw or ears, accompanied by mild to moderate fever, and symptoms of thyrotoxicosis with tenderness on palpation of the thyroid gland [1, 2]. The etiology of SAT is still unknown, and virus infections such as coxsackie virus, adenovirus and influenza virus were previously thought to be the predisposing factors of SAT [3]. A small number of atypical SAT cases without neck pain or tenderness have been reported in the literature. Thus, attention should be paid to SAT in clinical practice [4, 5].

Herein, we report a patient with simple acute headache who did not have typical thyroid pain and was misdiagnosed as meningitis. His headache symptoms were associated with SAT, and his symptoms gradually resolved with SAT treatment. At present, headache caused by Graves' disease and painless thyroiditis has occasionally been reported, but no detailed cases of headache caused by SAT have been reported.

## Case presentation

A 52-year-old male presented with headache for 1 week. The headache was mainly located in the occipital area with paroxysmal onset. The pain was milder in the morning and aggravated in the afternoon. The pain became progressively worse in the first week. The patient had low-grade fever (the highest temperature was 37.6 ℃) and no nausea, vomiting, and limb movement disorder. Nervous system physical examination showed that Kernig’s sign was suspiciously positive, and no other positive signs were found. The patient had a history of upper respiratory tract infection 2 weeks prior to onset. He had no history of other diseases and no history of long-term drug use.

When the patient was first admitted to another hospital, blood tests showed elevated C-reactive protein (CRP, 37.1 mg/L), normal white blood cell count (WBC, 5.3 × 10^∧^9/L) and magnetic resonance imaging of the brain showed no abnormalities. The doctor gave cefixime for anti-infection and ibuprofen for anti-inflammatory and analgesic treatment. After four days of treatment, the patient’s headache and fever symptoms were not relieved. Reexamination results on the fourth day showed that CRP was increased to 50.2 mg/L. The patient came to the neurology department of our hospital for further treatment. At admission, blood tests showed CRP levels of 64.8 mg/L (reference range, < 10.0 mg/L), normal leukocytes, and Erythrocyte sedimentation rate (ESR) of 68 mm/H (reference range, 0–15 mm/H). The patient had obvious headache symptoms, and the neurologist considered the possibility of meningitis by combining headache, fever, and suspicious positive Kirschner’s sign. Therefore, acyclovir combined with ceftriaxone was used for anti-infection and symptomatic relief of headache. The neurologist also performed a lumbar puncture to check for cerebrospinal fluid (CSF). The intracranial pressure was 215 mmH_2_O, the number of nucleated cells in cerebrospinal fluid was 0.006 × 10^∧^9/L(reference range, < 0.008 × 10^∧^9/L), the glucose level was 4.66 mmol/L(reference range, 2.70–4.20 mmol/L), and the protein level was 402.8 mg/L(reference range, 120.0–400.0 mg/L). Cryptococcus was not found in cerebrospinal fluid, and the result of bacterial culture was negative. No evidence of meningitis was found, and the patient’s headache and fever symptoms did not relieve after two days of treatment.

**Fig. 1:**
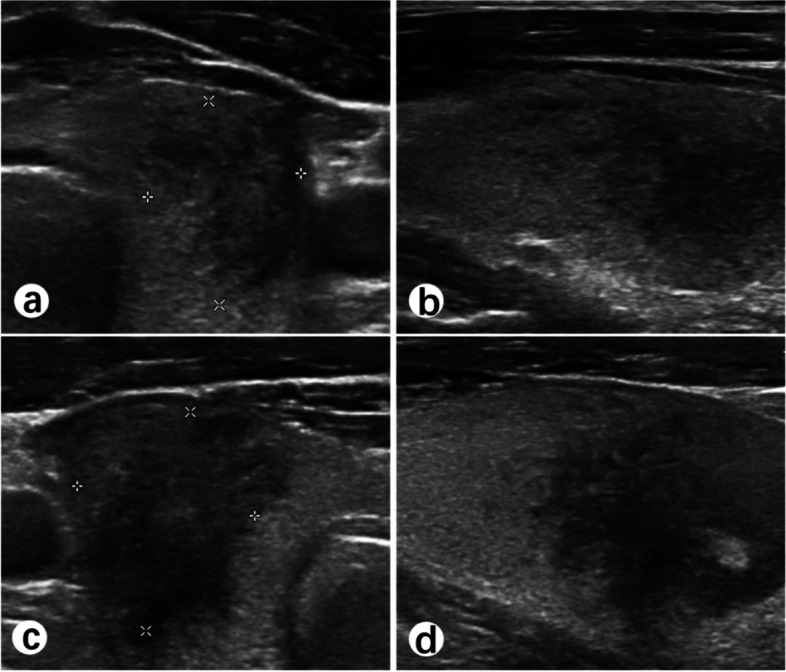
Diffuse hypoechoic areas of bilateral thyroid gland. Transverse view of the left thyroid gland (**a**). Longitudinal view of the left thyroid gland (**b**). Transverse view of the right thyroid gland (**c**). Longitudinal view of the right thyroid gland (**d**)

Other tests were performed, and the results showed thyrotoxicosis. Thyroid-stimulating hormone decreased to 0.046μIU/mL (reference range, 0.350–4.940 μIU/mL), free triiodothyronine (FT3) was 5.31 pg/mL (reference range, 1.58–3.91 pg/mL), free thyroxine (FT4) was 1.61 ng/dL (reference range, 0.70–1.48 ng/dL), and thyroglobulin was elevated to 151.20 ng/mL (reference range, < 77.00 ng/mL). Thyroid-stimulating hormone receptor antibody (TRAb), thyroid peroxidase antibody (TPOAb), and thyroglobulin antibody (TGAb) were negative. Further examination of thyroid color ultrasound showed bilateral diffuse hypoechoic areas (Fig. 1), and color Doppler ultrasound first suggested SAT. Thyroid examination was done again, and mild tenderness of the left thyroid was found. Finally, the diagnosis of SAT was made after consultation with an endocrinologist, and methylprednisolone 40 mg was given to the patient. After 1 day of treatment, the patient’s headache was relieved, and his body temperature returned to normal. ESR and CRP returned to normal after 2 weeks of methylprednisolone treatment. Thyroid function returned to normal after 1 month of treatment.

## Discussion and conclusions

SAT usually has a three-stage course lasting about 6 months. In the first few weeks, as the thyroid follicles are damaged, the stored thyroid hormone is released into the bloodstream, leading to transient thyrotoxicosis. Thyroglobulin is synthesized by thyroid follicular epithelial cells and secreted into the follicular lumen, and thyroid hormone is synthesized on thyroglobulin. When follicles are damaged, thyroglobulin is also released, causing its levels in the blood to rise. After the inflammation subsides, the disease enters the hypothyroidism stage, which may last for three months or more before returning to normal function [6]. TPOAb and TGAb tests are required to exclude Hashimoto’s thyroiditis in the diagnosis of SAT, and TRAb levels need to be determined to differentiate Graves’ disease as they differ in treatment [7, 8].

SAT usually presents with thyroid pain as the chief complaint, but this was not present in our case, which presented as a simple headache. The pain of subacute thyroiditis is mainly caused by inflammation. The stimulation of inflammatory factors, exudation of tissue fluid and elevation of basal metabolic rate can lead to thyroid tenderness. Neck pain may not be present in some types of thyroiditis, such as Hashimoto's thyroiditis and postpartum thyroiditis, both of which are associated with autoimmunity. Hashimoto's thyroiditis usually contains TPOAb and TGAb, and diffuse thyroid lesions can be seen in thyroid color ultrasound. Long-term development can lead to hypothyroidism, which is not consistent with our case. Postpartum thyroiditis is a lymphocytic inflammation of the thyroid that is self-limiting and usually occurs within 12 months of a woman giving birth [9]. Most of the patients with postpartum thyroiditis had no symptoms of viral infection before the onset of the disease, which was more common in areas with high dietary iodine intake [10]. Among the 11 patients with thyrotoxicosis who reported headache, 82% were female, with an average age of 38 years, and the average free T4 level was 7.5 ng/dL [11]. By contrast, our patient was male, older, had a history of prodromal infection, and significantly lower free T4 levels than these cases.

The goal of SAT treatment is to improve symptoms. Guidelines from the American Thyroid Association recommend Non-Steroidal Antiinflammatory Drugs (NSAIDs) for mild symptoms and glucocorticoids for severe or nonresponsive patients [12]. In our case, there was no remission of symptoms with NSAIDs and a rapid remission with glucocorticoids. Studies have shown that steroid hormones provide pain relief for a shorter period of time and can reduce the overall duration of the disease [13, 14]. Another study involving 295 SAT participants found that 60% of patients who received ibuprofen did not experience pain relief within 2 weeks, and that patients with clearly high ESR and CRP had a lower response rate to ibuprofen treatment [15]. Therefore, if the ESR level at diagnosis has increased approximately 2.5-fold and the CRP level has increased approximately fourfold, and the effect of ibuprofen treatment is not satisfactory, steroid therapy may be the first choice [15].

According to thyroid-related antibodies and our treatment results, the thyroid hormone itself causes headache in patients, not various antibodies. In our case, the patient’s headache symptoms were relieved with the improvement of thyrotoxicosis. Very little is known about thyroid hormone causing headaches. In rat experiments, thyroxine increased the concentration of lipid peroxides in tissues with high metabolic rate (including the brain), and decreased the levels of antioxidant enzymes and glutathione transferase in the cerebral cortex [16, 17]. High levels of lipid peroxides and low antioxidant enzymes also exist in human hyperthyroidism [18]. Therefore, Borkum believed that thyroxine could lead to increased oxidative stress in the brain and up-regulate cortical excitability by reducing the synthesis of γ-aminobutyric acid [19]. At the same time, thyroid hormone has sympathomimetic activity. Vasospasm and constriction caused by sympathetic tension can also be a cause of headache.

In addition, our patient’s CSF pressure indicates intracranial hypertension, which may also be the cause of his headache. Thyrotoxicosis may reduce cerebrovascular resistance and increase cerebral venous blood flow, leading to intracranial congestion and headache [20]. In addition, thyroxine assists in sodium delivery and can lead to dynamic changes in CSF. Increased intracranial pressure leads to increased central venous sinus pressure, and the compression of arachnoid villi leads to decreased CSF absorption, which can further increase CSF pressure [21].

In the diagnosis and treatment of this case, the patient had no previous history of headache, and we excluded migraine, tension headache, trigeminal neuralgia and other primary headaches. Through medical history, laboratory and imaging studies, we ruled out secondary headache factors such as medication, mental illness, radiation injury, meningitis, head trauma, cerebrovascular disease, and intracranial space occupation [22]. In addition, influenza syndrome can also present with headache, but the symptoms of influenza usually subside within 1 week [23]. This patient had an upper respiratory tract infection 2 weeks before the onset of the disease, and the symptoms of upper respiratory tract infection such as sore throat, nasal congestion, and runny nose disappeared when the headache appeared. So we don't think the headache is caused by the flu. In summary, the patient had subacute thyroiditis, thyroid function improved after glucocorticoid therapy, and headache was also relieved. Therefore, we believe that his headache is a secondary headache caused by changes in thyroid hormone levels in the body due to subacute thyroiditis.

In conclusion, this patient is the first detailed report of SAT presenting with simple headache. There is a correlation between thyrotoxicosis and headache. From the perspective of thyroid related antibodies and our treatment results, it is thyroid hormone itself, rather than various antibodies, that causes headache in patients. Therefore, changes in thyroid hormone should be detected during treatment and follow-up. The mechanism between headache and thyrotoxicosis is not clear. Thus, more studies are needed to help clinicians diagnose and treat headache in these cases.

## Data Availability

All data related to this report are kept at Huzhou Central Hospital, Affiliated Central Hospital of Huzhou University (Zhejiang Province, China), and are available from the corresponding author on reasonable request.

## Abbreviations

SAT: Subacute thyroiditis
CRP: C-reactive protein
WBC: White blood cell
ESR: Erythrocyte sedimentation rate
CSF: Cerebrospinal fluid
FT3: Free triiodothyronine
FT4: Free thyroxine
TRAb: Thyroid-stimulating hormone receptor antibody
TPOAb: Thyroid peroxidase antibody
TGAb: Thyroglobulin antibody
NSAIDs: Non-Steroidal Antiinflammatory Drugs
